# Fbxo45‐mediated NP‐STEP_46_ degradation via K6‐linked ubiquitination sustains ERK activity in lung cancer

**DOI:** 10.1002/1878-0261.13290

**Published:** 2022-08-05

**Authors:** Qian Wang, Ci Xu, Renjie Cai, Weishu An, Haihua Yuan, Ming Xu

**Affiliations:** ^1^ Department of Oncology, Shanghai Ninth People's Hospital Shanghai Jiao Tong University School of Medicine China; ^2^ Department of Gastroenterology, Shanghai Ninth People's Hospital Shanghai Jiao Tong University School of Medicine China

**Keywords:** drug resistance, ERK1/2, Fbxo45, NSCLC, STEP

## Abstract

Lung cancer is one of the most threatening malignant tumors to human health. Epidermal growth factor receptor (EGFR)‐targeted therapy is a common and essential means for the clinical treatment of lung cancer. However, drug resistance has always affected the therapeutic effect and survival rate in non‐small cell lung cancer (NSCLC). Tumor heterogeneity is a significant reason, yielding various drug resistance mechanisms, such as EGFR‐dependent or ‐independent extracellular signal‐regulated kinase 1 and/or 2 (ERK1/2) activation in NSCLC. To examine whether this aberrant activation of ERK1/2 is related to the loss of function of its specific phosphatase, a series of *in vitro* and *in vivo* assays were performed. We found that F‐box/SPRY domain‐containing protein 1 (*Fbxo45*) induces ubiquitination of NP‐STEP_46_ , an active form of striatal‐enriched protein tyrosine phosphatase, with a K6‐linked poly‐ubiquitin chain. This ubiquitination led to proteasome degradation in the nucleus, which then sustains the aberrant level of phosphorylated‐ERK (pERK) and promotes tumor growth of NSCLC. *Fbxo45* silencing can significantly inhibit cell proliferation and tumor growth. Moreover, NSCLC cells with silenced *Fbxo45* showed great sensitivity to the EGFR tyrosine kinase inhibitor (TKI) afatinib. Here, we first report this critical pERK maintenance mechanism, which might be independent of the upstream kinase activity in NSCLC. We propose that inhibiting *Fbxo45* may combat the issue of drug resistance in NSCLC patients, especially combining with EGFR‐TKI therapy.

## Graphical abstract

EGFR‐TKI‐targeted therapy is the most widely used clinical treatment for lung cancer; however, drug resistance remains a significant challenge. Here we investigate a molecular mechanism behind EGFR‐TKI drug resistance in patients with NSCLC. Inhibition of Fbxo45 protein stabilized ERK‐specific phosphatase NP‐STEP_46_ and increased sensitivity to EGFR‐TKI by attenuating the maintenance of pERK activity in NSCLC.

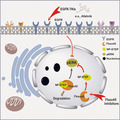

**Abbreviations**
BRAF: V‐Raf murine sarcoma viral oncogene homolog B
EGFR: epidermal growth factor receptor
ERK: extracellular signal‐regulated kinase
Fbxo45: F‐box/SPRY domain‐containing protein 1
GST: glutathione S transferase
IHC: immunochemistry
KIM: kinase interaction motif
KRAS: Kirsten rat sarcoma viral oncogene homolog
LUAD: lung adenocarcinoma
LUSC: lung squamous cell carcinoma
MAPK: mitogen‐activated protein kinase
MycBP2: MYC binding protein 2
NSCLC: non‐small cell lung cancer
NP‐STEP: non‐phosphorylated striatal‐enriched protein tyrosine phosphatase
SCLC: small cell lung cancer
SKP1: S‐phase kinase associated protein 1
TKI: tyrosine kinase inhibitor
TTF1: transcription termination factor 1

## 1 Introduction

Lung cancer remains the most common cancer disease and the leading cause of cancer‐related mortality, with an estimated 2.21 million new cases and 1.80 million deaths per year globally [1, 2]. Lung cancer is roughly divided into small cell lung cancer (SCLC, 15% of lung cancer) and non‐small cell lung cancer (NSCLC, 85% of lung cancer) in terms of the diverse etiologies [3, 4]. Both SCLC and NSCLC are closely associated with smoking [5]. Lung adenocarcinoma (LUAD), as a usual subtype of NSCLC, is also the most common histology in never smokers, as well as in women and young adults [5, 6], which could be associated with environmental exposures or inherited genetic susceptibility [5]. Despite significant advances in the diagnosis and therapy of lung cancer, the 5‐year relative survival rate is still only 6% in most patients (57%) diagnosed with metastatic events [4]. Epidermal growth factor receptor (EGFR, also known as ERBB1) is frequently mutated in NSCLC cells and has been the promised target for NSCLC treatment for the last decade [7]. With the iterative update of EGFR tyrosine kinase inhibitors (EGFR‐TKIs) and the treatment improvements, the 2‐year survival for NSCLC patients increased from 34% during 2009 through 2010 to 42% during 2015 through 2016 [4]. However, most individuals harboring wild‐type EGFR, KRAS/BRAF mutations, ALK/ROS1 rearrangements/fusion, or MET amplification still do not benefit from EGFR‐TKI therapy, which results in an unsatisfactory 5‐year survival rate [5, 8].

In NSCLC, the ERK1/2 is usually aberrantly activated and sustained the phosphorylation status, which has been thought to be the critical link with the events of tumor growth and malignant transformation [9, 10]. EGFR, as a tyrosine kinase, responds to the main ligands of epidermal growth factor (EGF), transforming growth factor α (TGF‐α), amphiregulin (AR), and epigen (EPG) stimulus, that mainly mediates the RAS–RAF–MEK–ERK and PI3K‐AKT–mTOR signaling pathway [11, 12]. EGFR‐TKIs are developed to suppress the abnormal signal transduction caused by EGFR mutations to treat NSCLC. The kinase cascade RAS–RAF–MEK–ERK has also been attempted for drug targets in clinical trials. Still, none of them has yielded satisfactory outcomes [13, 14, 15], particularly the KRAS inhibitor [16, 17] and the MEK inhibitor [18]. The activity of ERK1/2 can be regulated by specific kinases and phosphatases. In other words, the sustained activity of ERK1/2 in NSCLC cells could result from the aberrant deactivation of its specific phosphatase, in addition to that induced by the EGFR, KRAS, or BRAF mutation. Given that, blocking abnormal activation of pERK induced by the kinase cascade and co‐activating the specific phosphorylase activity to pERK may be a novel therapeutic strategy for further improving the survival rate of NSCLC patients.

Striatal‐enriched protein tyrosine phosphatase (STEP), a member of the brain‐rich and non‐receptor protein tyrosine phosphatases (PTPs), can bind to and inactivate ERK1/2 through kinase interaction motif (KIM) [19, 20, 21], which has also been confirmed in STEP knockout mice model [22]. The STEP protein encoded by the PTPN5 gene can generate two main functional isoforms, a membrane‐associated protein termed STEP_61_ and a cytosolic protein termed STEP_46_ [22, 23, 24]. In the past decades, scientists have revealed that STEP mainly enriches within the central nervous system (CNS) and involves alterations in synaptic transmission, plasticity, memory formation, and neurological disorders [19, 22, 25, 26, 27]. However, whether and how STEP phosphatase contributes to the occurrence and development of tumor‐associated diseases, particularly the influence on the therapeutic effect of chemotherapeutic agents (such as EGFR‐TKIs applied in lung cancer), is still in the puzzle. In the KIM domain of STEP, Serine 49 (S49) in STEP_46_ or Serine 221 (S221) in STEP_61_ is the primary residue for phosphorylation by cAMP‐dependent protein kinase A (PKA) to render STEP activity [28, 29]. Dephosphorylation of these residues leads to poly‐ubiquitination and proteolytic degradation of Non‐Phospho‐STEP (NP‐STEP) [29]. Several kinds of literature have reported that proteolytic degradation of STEP occurs upon the synaptic NMDA receptor activation in Schizophrenia [30] or the suppression of brain‐derived neurotrophic factor (BDNF) in neuropsychiatric disorders [31], as well as that does in long‐term potentiation and learning [32]. However, there is no report concerning specific E3 ubiquitin ligase in charge of STEP stability, except Parkin for membrane‐associated protein STEP_61_ in Parkinson's disease [33].

Here, we identify that Fbxo45, a component of the E3 ligase complex Skp1‐Pam‐Fbxo45 (SPF) [34, 35], ubiquitinates and induces proteolytic degradation of the cytosolic STEP_46_ via the K6‐ubiquitin linkage, thereby leading to the persistent activation of ERK. High‐expressed Fbxo45 in NSCLC cells enhances the malignant property. In contrast, ablating Fbxo45 maintains the STEP_46_ activity to abolish the aberrant ERK1/2 signaling, which may greatly serve the NSCLC treatment, especially by combining with EGFR‐TKIs, to offer a possible way to relieve EGFR‐TKI tolerance and improve the prognosis of NSCLC patients.

## 2 Materials and methods

### 2.1 Cell culture

Human non‐small cell lung cancer (A549, NCI‐H1299, NCI‐H1650, and NCI‐H1975), human cervical carcinoma (HeLa), and human embryonic kidney 293T (HEK293T) cell lines were purchased from the Cell Bank of Type Culture Collection of Chinese Academy of Sciences (Shanghai, China). NCI‐H1299, NCI‐H1650, and NCI‐H1975 cell lines were cultured in Roswell Park Memorial Institute (RPMI) 1640 medium (Hyclone, Logan, UT, USA) with 10% fetal bovine serum (BioSun, Shanghai, China), 100 mg·mL^−1^ streptomycin (BioSun), 100 U·mL^−1^ penicillin (Hyclone), and 1% GlutaMAX (Gibco, Grand Island, NY, USA). A549 and HEK293T cell lines were cultured in high‐glucose Dulbecco's modified eagle medium (DMEM; Hyclone) with the same supplements mentioned above. All cell lines used in this project were authenticated by STR identification and tested for mycoplasma contamination.

### 2.2 Antibodies and reagents

Antibodies against rabbit non‐phospho‐STEP (NP‐STEP; D74H3, #5659), HA‐tag (C29F4, #3724), p‐AKT (Ser473, 193H12, #4058), AKT (#9272), ERK (#4696), and pp38 (Thr180/Tyr182; D3F9, #4511) were all purchased from Cell Signaling Technology (Danvers, MA, USA). Donkey anti‐rabbit IgG Cy3 and IgG fraction monoclonal mouse anti‐rabbit IgG (light chain‐specific) antibodies were purchased from Jackson ImmunoResearch (West Grove, PA, USA). p38 (ab170099), goat anti‐rabbit IgG Alexa Fluor 568 (ab175471), goat anti‐mouse Alexa Fluor 488 (ab150077), and mounting medium with DAPI (ab104139) were purchased from Abcam (Cambridge, UK). Antibodies against mouse M2 Flag antibody (F1804), rabbit Fbxo45 (HPA040730), and MG132 (SML1135) were purchased from Sigma‐Aldrich (St. Louis, MO, USA). Antibodies against mouse p‐ERK1/2 (E4, sc‐7383), Ki‐67 (sc‐23 900), and Ubiquitin (P4D1, sc‐8017) were obtained from Santa Cruz (Santa Cruz, CA, USA). Puromycin (P8230) was purchased from Solarbio (Beijing, China). Afatinib (S1011), Osimertinib (S7297), and protease inhibitor cocktail (EDTA‐Free, 100× in DMSO) were purchased from Selleck (Houston, TX, USA). Ni^2+^‐NTA beads (#30210) were purchased from QIAGEN (Hilden, Germany). All primers synthesis and gene sequencing were conducted by Genewiz (Suzhou, China). The tissue microarray chips (Cat no. XT12‐033, Lot No. HLug‐Ade150CS‐01), with the information on pathological H&E staining, were obtained from Shanghai Outdo Biotech Company (Shanghai, China).

### 2.3 Plasmids

pGreenPuro™ shRNA lentiviral system (System Biosciences, La Jolla, CA, USA) was used for shRNA‐mediated knockdown of Fbxo45 in the cells. The shRNA sequences are followed as below: sh1 F: 5′GATCCGGAGGAGAGATCTGCTTATGGCTCGAGCCATAAGCAGATCTCTCCTCCTTTTTG3′; sh1 R: 5′AATTCAAAAAGGAGGAGAGATCTGCTTATGGCTCGAGCCATAAGCAGATCTCTCCTCCG3′; sh2 F: 5′GATCCGGGCCTTCATTTACGTAAATTCTCGAGAATTTACGTAAATGAAGGCCCTTTTTG3′; sh2 R: 5′AATTCAAAAAGGGCCTTCATTTACGTAAATTCTCGAGAATTTACGTAAATGAAGGCCCG3′. Mutations of Fbxo45 or STEP were generated by using the KOD‐plus‐mutagenesis kit (TOYOBO, Osaka, Japan) and Exnase II enzyme (Vazyme, Nanjing, China) system according to the manufacturer's instructions. Cell transfection was performed using Hiff‐Trans™ Liposomal Transfection Reagent (YEASEN, Shanghai, China). To generate Fbxo45 knockout cell lines, The sgRNA (5′CACCGGCGATGAGAACAGCGAGGTG3′) targeting Fbxo45 was cloned into the restriction endonuclease sites BbsI in pSpCas9 (BB)‐2A‐GFP vector (PX458, Addgene). After transfection for 36 h, GFP‐positive cells were screened by flow cytometer (Becton Dickinson, Franklin Lakes, NJ, USA) and then subjected to single‐cell isolation and expansion to select the positive cell clone.

### 2.4 Soft agar colony formation assay

The designated cells were seeded into soft agar to determine the ability of colony formation as described previously [36]. Briefly, 0.6% base agar gel (Amresco, Houston, TX, USA) in 2 mL medium was solidified as the lower layer in the 6‐well plates. The 2 × 10^3^ cells were seeded into a 2 mL medium containing 0.35% base agar gel and 5% FBS and layered onto the top layer. After 2 or 3 weeks, developed cell colonies were stained with 0.02% crystal violet in 20% methanol. The images of colonies were captured under an inverted microscope (Nikon TE2000‐U, Tokyo, Japan), and the number of colonies was counted for statistical analysis using imagej software (NIH, Bethesda, MD, USA).

### 2.5 Immunoprecipitation

The HEK293T cells or stable‐transfected cells were lysed in RIPA buffer I (150 mm NaCl, 50 mm Tris–HCl pH 7.4, 1% NP‐40, 1× proteasome inhibitor cocktail). Lysates were incubated with specific antibodies and appropriate Pierce™ Protein A/G magnetic beads (Waltham, MA, USA) at 4 °C overnight. After washing with the RIPA buffer I three times, the immunoprecipitates were resolved in SDS/PAGE and subjected to western blot analysis.

### 2.6 Immunochemistry (IHC)

Staining of Fbxo45, NP‐STEP, and pERK was performed as previously described [37] using rabbit polyclonal anti‐NP‐STEP (1 : 50), anti‐Fbxo45 (1 : 50), or mouse monoclonal anti‐pERK (1 : 20). The following secondary antibodies, HRP labeled goat anti‐rabbit (Servicebio, Shanghai, China), were used at 200‐fold dilutions. Donkey anti‐rabbit IgG Cy3 antibody (1 : 200) was used for the immunofluorescence staining in paraffin‐embedded tissues of NSCLC. Images were captured using the microscope (Nikon TE2000‐U). The IHC data were blindly analyzed and scored by three independent pathologists. The intensity was scored as follows: 0, negative; 1, weak; 2, moderate; and 3, strong. The frequency of positive cells was defined as follow: 0, less than 5%; 1, 5–25%; 2, 26–50%; 3, 51–75%; and 4, greater than 75%. The score is the sum of the intensity times the frequency in heterogeneous tumor samples.

### 2.7 Immunofluorescence

Monolayer HeLa cells with 50% confluence on slides were fixed in 4% paraformaldehyde for 10 min at room temperature and washed three times with cold PBS. The cells were treated with 0.1% TritonX‐100 in PBS for 10 min and blocked with 5% BSA/PBST for 30 min at room temperature. After three times washing, the cells were incubated with the primary antibodies diluted in 1% BSA/PBST at 4 °C overnight. The goat anti‐rabbit Alexa Fluor 568 or goat anti‐mouse Alexa Fluor 488 were applied in the dark for 1 h at room temperature. After washing, the slides were sealed with DAPI (0.5 ng·mL^−1^) solution for image capture under the laser scanning confocal microscope (Leica Biosystems, Deer Park, IL, USA).

### 2.8 GST protein pull‐down assay

*Escherichia coli* BL21 expressing pGEX‐6p‐1‐Fbxo45 or pGEX‐6p‐1 vector was lysed in B‐PER Protein Extraction Reagent (#78248, Thermo Fisher Scientific, Waltham, MA, USA) according to the instruction. Lysates were incubated with Glutathione sepharose 4B (Thermo Fisher Scientific, Waltham, MA, USA) overnight at 4 °C and washed thrice with lysis buffer. HEK293T cells expressing Flag‐tagged STEP or its mutants were lysed in RIPA buffer II (150 mm NaCl, 50 mm Tris–HCl pH 7.4, 1% Triton X‐100, 0.5% sodium deoxycholate, 1 mm EDTA, 5% glycerol, 1× proteasome inhibitor cocktail). Then, lysates were incubated with purified GST‐tagged Fbxo45 overnight at 4 °C. After three times washing, the precipitations were resolved in SDS/PAGE and subjected to western blot analysis.

### 2.9 Animal xenograft

The SPF grade Balb/c nude mice were obtained from Shanghai JieSiJie Laboratory Animal Co., Ltd (License No. SCXK(Hu)2018–0004) (Shanghai, China). Four‐week‐old female nude mice with similar weight, size, and healthy state were divided into required groups randomly, and at least five mice in each group were guaranteed after potential loss. The mice were bred in a specific pathogen‐free condition (temperature: 22–25 °C, and humidity: 40%). 1 × 10^6^ A549 cells or H1975 cells in 100 μL PBS were subcutaneous injected into each mouse. At about 10 days, the grouped mice harboring primary tumors xenografted from H1975 cells were sub‐grouped for intragastric administration with DMSO or Afatinib (10 mg·kg^−1^). The mice in each group were monitored for tumor volume measuring every 4 days and sacrificed at 27 days post‐injection. After the mice were ethically executed under the condition of anesthesia using 1% pentobarbital sodium following Chinese Laws and Requirements on Experimental Animal, the xenografted tumors were collected for weight measuring and fixed with 4% paraformaldehyde for hematoxylin and eosin (H&E) staining and immunohistochemistry detection. Mice that died or were injured due to biting were excluded from the statistical analysis. The ethics committee approved this study involving animals for experiments research at Shanghai Ninth People's Hospital, Shanghai Jiao Tong University School of Medicine (Number: HKDL[2016]56).

### 2.10 Statistical analysis

Each presented cell experiment was set in duplicate or triplicate and performed at least three times for the power analysis. The significance of the difference and variation (mean ± SD or mean ± SEM) was calculated and tested with a two‐tail non‐paired or paired *t*‐test by using graphpad prism software (GraphPad Software, San Diego, CA, USA), in which *P* < 0.05 (*), < 0.01 (**), or < 0.001 (***) was considered statistically significant.

## 3 Results

### 3.1 Aberrant Fbxo45 expression is probably associated with the development of NSCLC

**Fig. 1:**
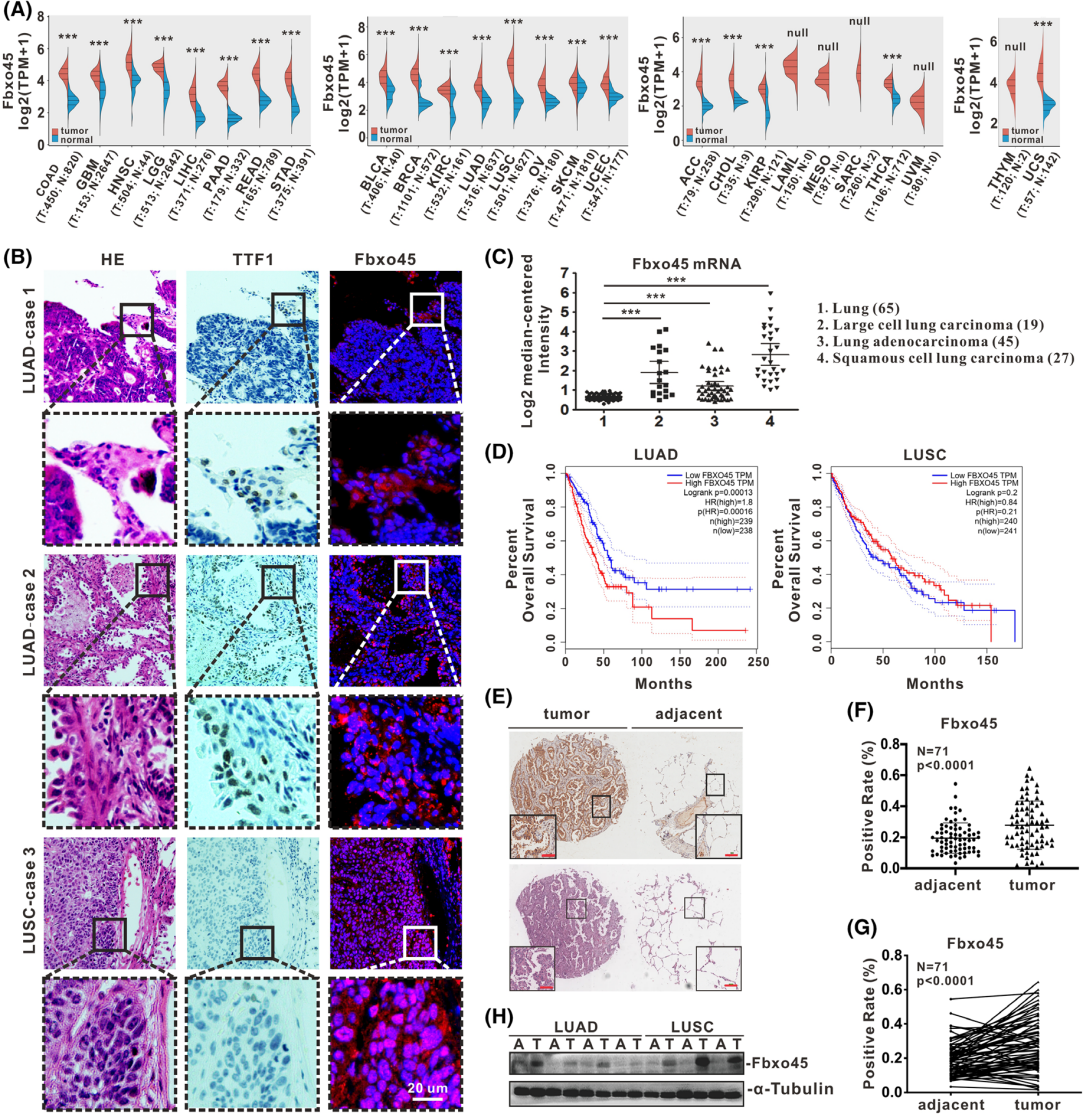
Aberrant Fbxo45 expression is probably associated with the development of NSCLC. (A) Expression distribution of the Fbxo45 gene in tumor and normal tissues was generated from the website (www.aclbi.com) based on TCGA and GTEx datasets. The significance of the two groups of samples passed the Wilcox test, and the asterisk represents the degree of importance (***P < 0.001). TPM, Transcripts per million; Null, Incomparable; T, Tumor; N, Normal; the case number was shown in the brackets. (B) The indirect immunofluorescence was used for Fbxo45 detection in the paraffin sections from NSCLC patients (right panel). The immunochemistry staining was used for TTF‐1 detection (middle panel), and HE staining was shown in the same scope (left panel). Three individual replications were set, and the representative images were reported. Scale bar: 20 μm. (C) The Fbxo45 expression was dot‐plotted and compared among normal lung, large cell lung carcinoma, lung adenocarcinoma, and squamous cell lung carcinoma according to the ONCOMINE TCGA dataset (https://www.oncomine.org). The case number was shown in the bracket in each group. The data are presented as mean ± SD with *t*‐test analysis, ***P < 0.001. (D) The GEPIA web tool (http://gepia.cancer‐pku.cn) was used to generate the survival curve for LUAD and LUSC patients based on Fbxo45 expression in TCGA datasets. The 95% confidence interval (CI) was set as dotted line. (E, F) the Fbxo45 protein level was detected by immunohistochemistry in the microarray tissue chip containing 75 pairs of LUAD patients, and the representative samples were presented with Fbxo45 detection and HE staining (E) scale bar: 100 μm. The positive incidence of Fbxo45 staining from 71 pairs of LUAD samples was statistically calculated using the software of aperio imagescope (Leica Biosystems) and dot‐plotted in pairs (G) or non‐pairs (F) with a *t*‐test by graphpad prism 8 (GraphPad Software Inc.). The data are presented as mean ± SD. (H) The fresh surgical resections from NSCLC patients were lysed in 2% SDS lysis buffer for immunoblotting. Two individual replications were set, and the representative images were reported.

As a conserved F‐box protein, Fbxo45 usually does not express in the lung or other non‐brain tissues [34]. According to the Pan‐cancer analysis, Fbxo45 transcripts are significantly enhanced in tumors compared with normal tissues, except those cancer types with no standard control (Fig. 1A). The forest plot for overall survival (OS) in Pan‐cancer also showed that Fbxo45 expression is significantly related to the hazard ratio in NSCLC, as well as brain low‐grade glioma (LGG), liver hepatocellular carcinoma (LIHC), and mesothelioma (MESO; Table 1). Intriguingly, Fbxo45 was specifically and strongly expressed in tumor cells of NSCLC according to the indirect immunofluorescence analysis in paraffin‐embedded human lung carcinoma, including LUAD with TTF1‐positive and LUSC with TTF1‐negative (Fig. 1B). To determine Fbxo45 mRNA expression in lung cancer, we used the ONCOMINE database to reveal that the relative mRNA levels of Fbxo45 were significantly higher in various types of human lung cancer than those in normal lung tissues (Fig. 1C). However, the overall survival curve indicated that the higher mRNA level of Fbxo45 is correlated with poor survival percentage in LUAD but not in LUSC patients (Fig. 1D). Likewise, a tissue microarray chip including 71 pairs of LUAD and adjacent tissues was examined by immunohistochemistry (Fig. 1E–G and Fig. S1A), which indicated that Fbxo45 protein expression is markedly increased in lung cancer tissues compared with the normal adjacent lung tissues (Fig. 1E–H). Consistent with that, seven pairs of NSCLC patients' fresh surgical specimens (four pairs of LUAD, three pairs of LUSC) revealed a similar outcome according to the western blot analysis (Fig. 1H). Together, these results drove the hypothesis that high‐expression of Fbxo45 is probably associated with the development of NSCLC.

**Table 1:**
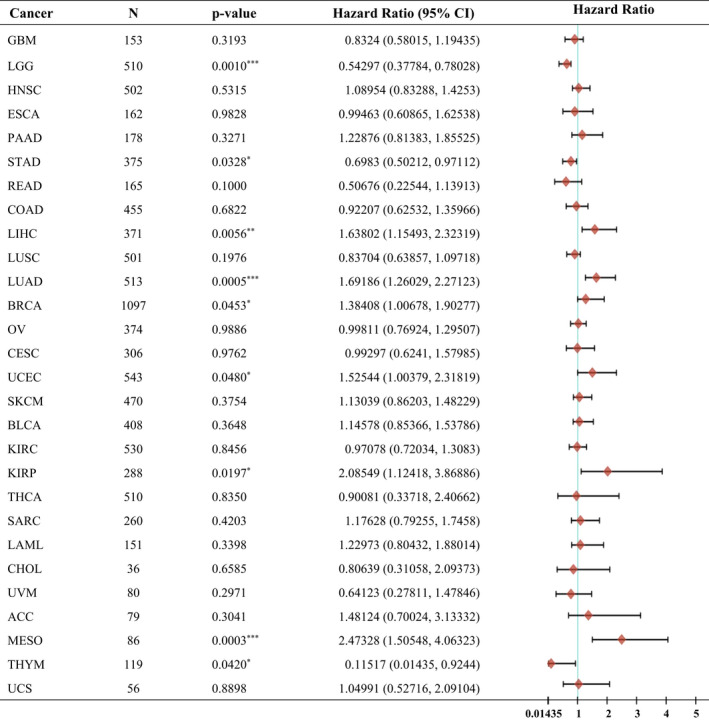
Hazard ratio involved in Fbxo45 univariate Cox regression in pan‐cancers. The ‘forestplot’ r package was used for univariate cox regression analysis and forest plotting to display each variable's *P*‐value, HR, and 95% CI. The rank‐sum test detects two sets of data, and a *P*‐value of < 0.05 is considered statistically significant. *P < 0.05, **P < 0.01, ***P < 0.001. ACC, Adrenocortical Carcinoma; BLCA, Bladder Urothelial Carcinoma; BRCA, Breast Cancer; CESC, Cervical Squamous Cell Carcinoma; CHOL, Cholangiocarcinoma; COAD, Colon Adenocarcinoma; ESCA, Esophagus Squamous Cell Carcinoma; GBM, Glioblastoma Multiforme; HNSC, Head and Neck Squamous Carcinoma; KIRC, Kidney Renal Clear Cell Carcinoma; KIRP, Kidney Renal Papillary Cell Carcinoma; LAML, Acute Myeloid Leukemia; LGG, Lower Grade Glioma; LIHC, Liver Hepatocellular Carcinoma; LUAD, Lung Adenocarcinoma; LUSC, Lung Squamous Cell Carcinoma; MESO, Mesothelioma; *N*, case number; OV, Ovarian serous cystadenocarcinoma; PAAD, Pancreatic Adenocarcinoma; READ, Rectum Adenocarcinoma; SARC, Sarcoma; SKCM, Skin Cutaneous Melanoma; STAD, Stomach Adenocarcinoma; THCA, Thyroid Carcinoma; THYM, Thymoma; UCEC, Uterine Corpus Endometrial Carcinoma; UCS, Uterine Carcinosarcoma; UVM, Uveal Melanoma.

### 3.2 Silencing Fbxo45 inhibits the proliferation of NSCLC cells and tumor growth in mice

**Fig. 2:**
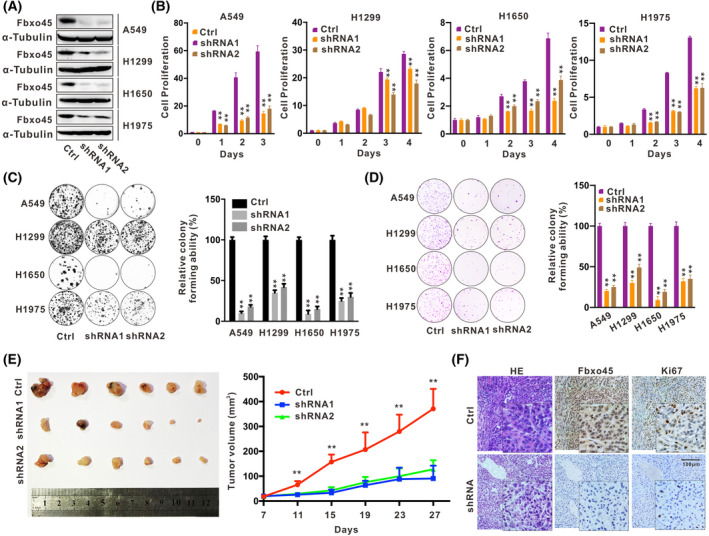
Silencing Fbxo45 inhibits the proliferation of NSCLC cells and tumor growth in mice. (A) Fbxo45 expression was detected by western blotting in the indicated cell lines infected and selected using the shRNA lentiviral system with three independent replications. (B) The proliferation of cell lines with or without Fbxo45 shRNA expression was presented in columns according to the CCK8 assay and statistically analyzed by graphpad prism 8 software. Data are presented as mean ± SEM with *t*‐test from three independent experiments. **P < 0.01. (C) The colony formation of cells was stained with 0.02% crystal violet in 12‐well plates (left panel) and statistically analyzed according to the positive‐stained cell number (right panel, data are presented as mean ± SEM with *t*‐test from three independent experiments. **P < 0.01). (D) In the anchorage‐independent growth assay, cells with or without Fbxo45 shRNA expression were stained with 0.05% crystal violet in 0.35% soft agarose (left panel) and statistically analyzed according to the positive‐stained cell colonies (right panel, data are presented as mean ± SEM with *t*‐test from three independent experiments. **P < 0.01). (E) The tumors were resected and compared from subcutaneous xenografted Balb/c nude mice at 27 days post‐injection with A549 cell lines with or without Fbxo45 shRNA expression (left panel). The tumor size was collected and compared in different periods, and data are presented as mean ± SEM with *t*‐test from three independent experiments. **P < 0.01 (right panel). (F) Fbxo45 and Ki67 were detected by H&E staining or immunohistochemistry detection in tumors from xenografted mice with A549 cells with or without Fbxo45 shRNA expression. Three individual replications were set, and the representative images were reported. Scale bar: 100 μm. [Colour figure can be viewed at wileyonlinelibrary.com]

To investigate the underlying biological function of Fbxo45, we generated stable cell lines with silenced Fbxo45 by using the lentiviral shRNA system in the four NSCLC cell lines (Fig. 2A). Fbxo45 depletion significantly inhibited the capabilities of cell proliferation (Fig. 2B) and colony formation on the cell‐culture plates (Fig. 2C). Likewise, the anchorage‐independent growth assay revealed the consistent outcomes that Fbxo45 promoted tumor growth in the soft agar (Fig. 2D). In the subcutaneously xenografted nude mice, A549 cells with abrogated Fbxo45 were significantly attenuated for tumor growth compared with the control cells with ‘normal’ Fbxo45 expression (Fig. 2E and Fig. S1B). Moreover, the IHC detection of the proliferation‐related Ki‐67 antigen also showed a supportive outcome (Fig. 2F). Together, these results demonstrated that Fbxo45 could promote cell proliferation and the tumorigenesis of NSCLC *in vitro* and *in vivo*.

### 3.3 Fbxo45 activates ERK by suppression of NP‐STEP_46_ in NSCLC cells

**Fig. 3:**
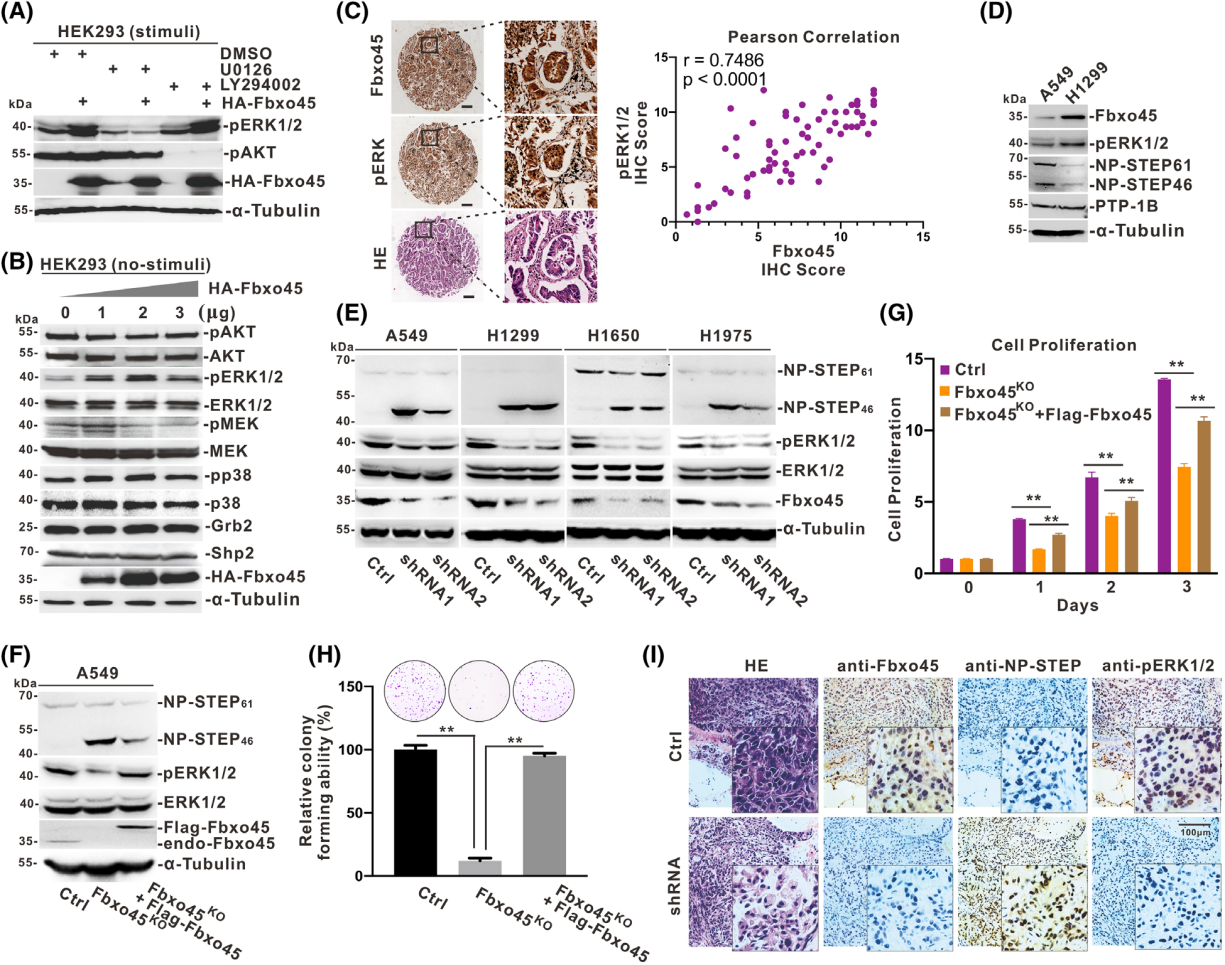
Fbxo45 activates ERK by suppression of NP‐STEP_46_ in NSCLC cells. (A) HEK‐293T cells transiently transfected with or without HA‐Fbxo45 plasmid for 48 h were treated with the fresh complete cell culture medium containing DMSO, U0126 (20 μm), or LY294002 (50 μm) for 1 h. The extracted cell lysis was resolved in SDS/PAGE gel for western blotting with antibody pERK, pAKT, HA, and tubulin as the loading control. Two individual replications were set, and the representative images were reported. (B) HEK‐293T cells were transiently transfected with a gradient amount of HA‐Fbxo45 plasmid for 48 h, and the cell lysis was resolved in SDS/PAGE gel for immunoblotting with the indicated antibodies. Two individual replications were set, and the representative images were reported. (C) Three independent pathologists estimated and scored the IHC score of Fbxo45 and pERK staining from 71 pairs of samples. The expression relation of Fbxo45 and pERK were statistically analyzed with Pearson correlation, the 95% CI was dotted (right panel). The representative images for IHC detection of Fbxo45 and pERK, along with HE staining, were presented in the left panel. Scale bar: 100 μm. (D) The cultured A549 and H1299 cells were harvested and lysed in RIPA buffer (150 mm NaCl, 50 mm tris–HCl pH7.4, 1% NP‐40, 0.1% SDS, 1% sodium deoxycholate, 1 mm EDTA, 1× proteasome inhibitor cocktail) for immunoblotting with the indicated antibodies. Two individual replications were set, and the representative images were reported. (E) The constructed cell lines with or without Fbxo45 shRNA expression were harvested and lysed in RIPA buffer for western blotting with the indicated antibodies. Two individual replications were set, and the representative images were reported. (F) The A549 cell was used to generate the Fbxo45‐knockout cell line (A549/Fbxo45^KO^) by the CRISPR‐Cas9 system, and the wide‐type A549 (A549/WT) cell line was set as a negative control. 3xFlag tagged Fbxo45 was re‐introduced into A549/Fbxo45^KO^ cells to generate A549/Fbxo45^KO^ + flag‐Fbxo45 cell line by the lentiviral system, and the A549/Fbxo45^KO^ and A549/WT infected with the control lentivirus were set as the system controls. A549/Fbxo45^KO^, A549/Fbxo45^KO^ + flag‐Fbxo45, and A549/WT cells were harvested and lysed in RIPA buffer for immunoblotting with the indicated antibodies. Two individual replications were set, and the representative images were reported. (G) A549/Fbxo45^KO^, A549/Fbxo45^KO^ + flag‐Fbxo45, and A549/WT cells were seeded in 96‐well plates (1000 cells per well) for the CCK8 assay. Data are presented as mean ± SEM with *t*‐test from three independent experiments. **P < 0.01. (H) A549/Fbxo45^KO^, A549/Fbxo45^KO^ + flag‐Fbxo45, and A549/WT cells were seeded in 6‐well plates (2000 cells per well) for the anchorage‐independent growth assay, and cell colonies were numbered and statistically columned. Data are presented as mean ± SEM with *t*‐test from three independent experiments. **P < 0.01. (I) Fbxo45, NP‐STEP, and pERK were detected by H&E or immunohistochemistry staining in tumors from xenografted mice with A549 cells with or without Fbxo45 shRNA expression. Three individual replications were set, and the representative images were reported. Scale bar: 100 μm. [Colour figure can be viewed at wileyonlinelibrary.com]

An interesting phenomenon has caught our attention while assessing the function of Fbxo45 in NSCLC cells. Fbxo45 was explicitly associated with the ERK but not AKT activity when overexpressed in HEK293T cells (Fig. 3A,B). The pERK level still can be sustained even though the MEK activity was quiescent (Fig. 3B). In addition, the other effect molecules closely associated with the ERK1/2 signal, including the phosphatase Shp2, which is supposed to be a prospective target for lung cancer therapy [38, 39, 40], showed no significant change upon the Fbxo45 expression (Fig. 3B). By the IHC detection in LUAD tissue microarray chips, the analyzed data showed a significant positive correlation between Fbxo45 and pERK (Fig. 3C and Fig. S1A,C). All of these drove us to consider whether Fbxo45 is related to the activity of specific phosphatases to ERK. Therefore, by comparing A549 and H1299 cell lines, we noted that the phosphatase STEP but not PTP‐1B was negatively associated with Fbxo45 and pERK levels (Fig. 3D). We tried to overexpress the cytoplasmic protein STEP_46_ in H1299 cells and confirmed that STEP protein could effectively inhibit the phosphorylation level of ERK1/2 and significantly suppress the proliferation of H1299 cells (Fig. S1D,E). To further verify the possibility of Fbxo45 affecting the STEP activity, knockdown Fbxo45 significantly enhanced the NP‐STEP level and correspondingly inhibited ERK activity in all four NSCLC cell lines (Fig. 3E). It is worth noting that Fbxo45 seemed only to affect the isoform STEP_46_ but not the isoform STEP_61_ (Fig. 3E,F).

To deeply inquire about the relationship among Fbxo45, NP‐STEP, and pERK, we established the Fbxo45 knockout cell line A549/Fbxo45^KO^ with the CRISPR‐Cas9 system. Furtherly, 3xFlag tagged Fbxo45 was re‐introduced into the KO‐cells using the lentiviral system and reactivated the suppressed pERK due to Fbxo45‐abortion (Fig. 3F). Consistent with that, the tumor cell proliferation and colony formation were also rescued by re‐applied Fbxo45 (Fig. 3G,H). In addition, the high Fbxo45 expression was distinctly alone with the high pERK level and the low NP‐STEP level and vice versa according to the H&E and IHC detection of the xenograft tumor samples from mice (Fig. 3I). Together, these results revealed that Fbxo45 could substantively activate ERK to promote tumor development by suppressing the NP‐STEP_46_ in NSCLC cells.

### 3.4 Fbxo45 mediated NP‐STEP_46_ proteasomal degradation via K6 ubiquitin linkage

**Fig. 4:**
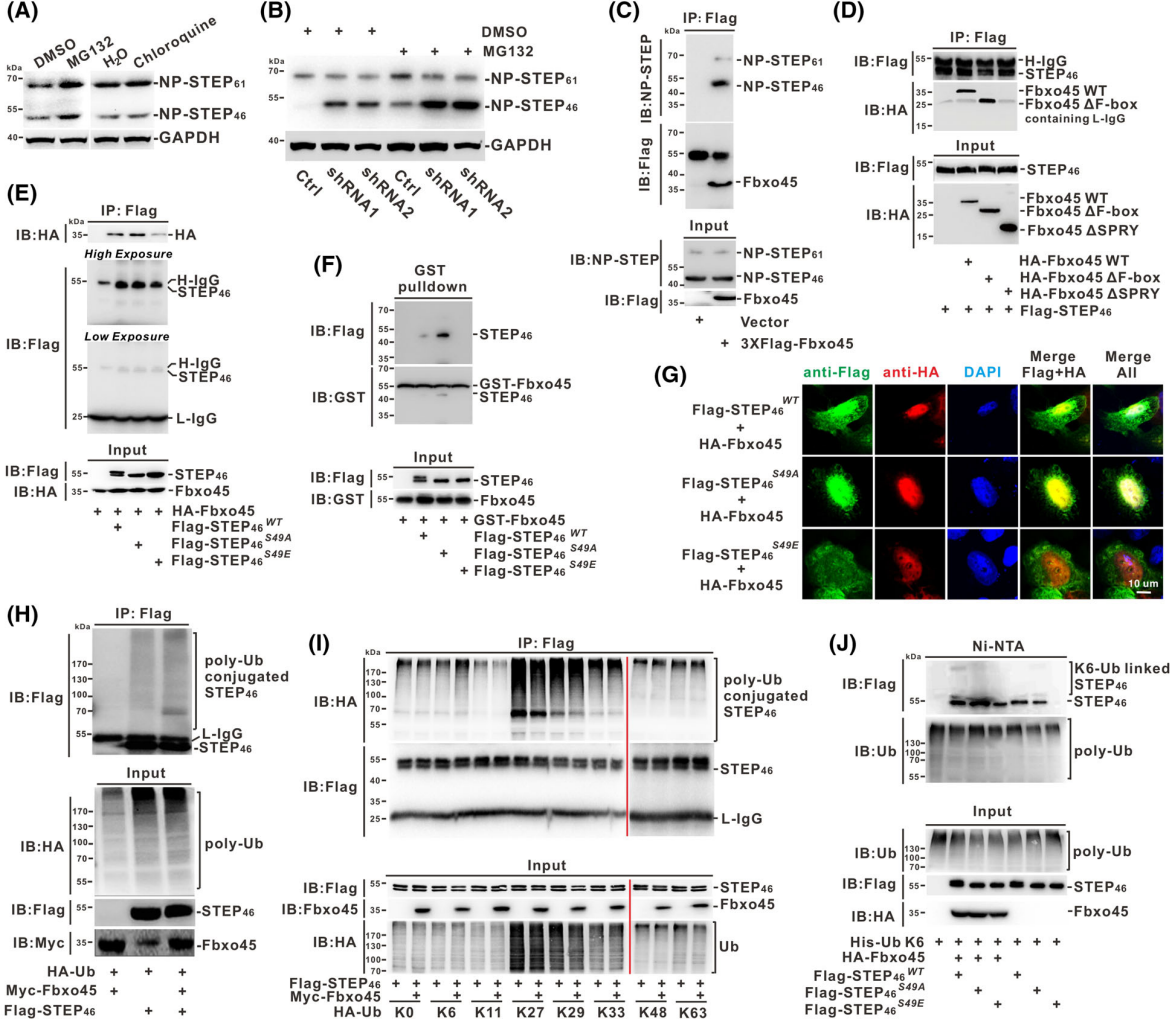
Fbxo45 mediated NP‐STEP_46_ proteasomal degradation via K6 ubiquitin linkage. (A) The A549 cells treated with MG132 (40 μm), chloroquine (20 μm), or vehicle for 4 h were harvested and lysed in RIPA buffer for immunoblotting with NP‐STEP or GAPDH antibody. Two individual replications were set, and the representative images were reported. (B) The A549 cells with or without Fbxo45 shRNA expression were harvested and lysed in RIPA buffer for immunoblotting with NP‐STEP or GAPDH antibody after MG132 (40 μm) or DMSO treatment for 4 h. Two individual replications were set, and the representative images were reported. (C) The A549 cells stably expressing 3xFlag‐Fbxo45 and control cells were treated with MG132 for 4 h before extraction. A total of 500 ng protein each was immunoprecipitated with 1 μg Flag M2 Mouse antibody in RIPA buffer II at 4 °C overnight. The washed immunoprecipitates were resolved in SDS/PAGE and immunoblotted with indicated antibodies. Two individual replications were set, and the representative images were reported. (D) Flag‐tagged STEP_46_ was co‐transfected into HEK293T cells with HA‐tagged Fbxo45 WT, Fbxo45 ΔF‐box, or Fbxo45 ΔSPRY, respectively. After 48 h transfection, the cell lysis in RIPA buffer II was immunoprecipitated with Flag M2 Mouse antibody and resolved for immunoblotting. Two individual replications were set, and the representative images were reported. (E) HA‐tagged Fbxo45 was co‐transfected into HEK293T cells with Flag‐tagged STEP_46_^WT^, STEP_46_^S49A^, or STEP_46_^S49E^. After 48 h of transfection, the cell lysis in RIPA buffer II was immunoprecipitated with Flag M2 Mouse antibody and resolved for immunoblotting. Two individual replications were set, and the representative images were reported. (F) GST‐tagged Fbxo45 was expressed in *E. coli* BL21 (DE3) and lysed with B‐PER protein extraction reagent (ThermoFisher) for purification with glutathione Sepharose 4B (ThermoFisher) at 4 °C overnight. The lysates from RIPA buffer II from HEK293T cells transfected with flag‐tagged STEP_46_^WT^, STEP_46_^S49A^, or STEP_46_^S49E^ were co‐cultured with purified GST‐Fbxo45, respectively, and the *in vitro* interaction was validated by immunoblotting with indicated antibodies. Two individual replications were set, and the representative images were reported. (G) The HeLa cells were co‐transfected with HA‐tagged Fbxo45 and Flag‐tagged STEP_46_^WT^, STEP_46_^S49A^, or STEP_46_^S49E^, respectively. The indirect immunofluorescence was conducted with the primary antibody Flag M2 Mouse (1 : 200) or HA rabbit (1 : 200). The second antibodies of goat anti‐rabbit IgG H&L Alexa Fluor 568 or goat anti‐mouse H&L Alexa Fluor 488 were used to detect the fluorescence signal under the laser scanning confocal microscope (Leica Biosystems). The cell nucleus was stained with DAPI. Scale bar:10 μm. Three individual replications were set, and the representative images were reported. (H) The lysis from HEK293T cells co‐transfected with HA‐tagged ubiquitin, Myc‐tagged Fbxo45, and Flag‐tagged STEP_46_ for 48 h was immunoprecipitated with Flag M2 Mouse antibody and immunoblotted with indicated antibodies. Two individual replications were set, and the representative images were reported. (I) HA‐tagged ubiquitin harboring with only one or no lysine (Kn or K0) was co‐transfected with Myc‐Fbxo45 and Flag‐STEP_46_ in HEK293T cells for 48 h. The lysates from RIPA buffer I was immunoprecipitated with Flag antibody and resolved in SDS/PAGE for immunoblotting. Two individual replications were set, and the representative images were reported. (J) His‐tagged Ubiquitin K6 was co‐transfected into HEK293T cells with Flag‐STEP_46_^WT^, ‐STEP_46_^S49A^, or STEP_46_^S49E^, respectively, along with or without HA‐Fbxo45 for 48 h. cells were lysed for precipitation with Ni^2+^‐NTA resin and then analyzed by immunoblotting with indicated antibodies. Two individual replications were set, and the representative images were reported. [Colour figure can be viewed at wileyonlinelibrary.com]

Since Fbxo45 is a crucial component of the ubiquitin E3 complex [34, 35], we hypothesized that suppressing NP‐STEP_46_ induced by Fbxo45 might relate to proteasomal degradation. To identify this, we treated A549 cells with the proteasome inhibitor MG‐132 or lysosome inhibitor Chloroquine (CQ) for western blotting analysis of the NP‐STEP stability. As shown, MG‐132 but not CQ stabilized both NP‐STEP_46_ and NP‐STEP_61_ protein (Fig. 4A), which is consistent with the reported literature that dephosphorylation of STEP leads to its ubiquitin‐mediated proteasomal degradation [29, 30, 32]. In the Fbxo45‐silenced cells, MG‐132 treatment could further enhance the level of NP‐STEP protein, particularly NP‐STEP_46_ (Fig. 4B). To assess the direct interaction between Fbxo45 and NP‐STEP, the semi‐endogenous immunoprecipitation assay was carried out in A549 stable cell line with 3xFlag Fbxo45 expression and showed that Flag‐tagged Fbxo45 could strongly interact with the endogenous NP‐STEP_46_ (Fig. 4C). In the structure of Fbxo45, the SPRY domain is supposed to be in charge of recognizing the specific substrates [34, 35, 41]. In line with that, the co‐immunoprecipitation assay revealed that Fbxo45 without SPRY domain (i.e., Fbxo45ΔSPRY) lost the ability of binding to STEP_46_ protein (Fig. 4D).

To identify whether Fbxo45 is more likely to recognize the active STEP, both the co‐immunoprecipitation and GST‐pull down assays proved the interaction of Fbxo45 with NP‐STEP_46_ and concluded the active form of NP‐STEP_46_ (S49A) could bind with Fbxo45 at most, while the inactive form of NP‐STEP_46_ (S49E) weakly bound to Fbxo45 (Fig. 4E,F). Consistently, the immunofluorescence assay reached similar outcomes that the active NP‐STEP_46_ (S49A) co‐localized with Fbxo45 mainly in the nucleus, whereas the inactive NP‐STEP (S49E) was more inclined to disperse in the cytoplasm (Fig. 4G). Since F‐box proteins play in recruiting their targeted substrates for further ubiquitination, Fbxo45 promoting the ubiquitylation of STEP_46_ is expected (Fig. 4H). To determine which kind of ubiquitin linkage leads to the degradation of NP‐STEP_46_, we applied mutants of HA‐tagged Ubiquitin in which remained none or only one of the seven lysine sites (K0, K6, K11, K27, K29, K33, K48, or K63) for ubiquitination assay and found that Fbxo45 specifically increased the K6‐linked ubiquitin chain on STEP_46_ (Fig. 3I). Ni‐NTA pull‐down assay also confirmed that K6‐ubiquitin linkage could conjugate on STEP protein, and Fbxo45 could promote the K6‐ubiquitination on STEP_46_, especially on its active form (Fig. 3J). Taken together, Fbxo45 can recognize the active STEP_46_ in the nucleus and induce the proteasome‐dependent degradation of NP‐STEP_46_ via K6 ubiquitin linkage.

### 3.5 Fbxo45 silencing increases the sensitivity to Afatinib on H1975 *in vitro* and *in vivo*

**Fig. 5:**
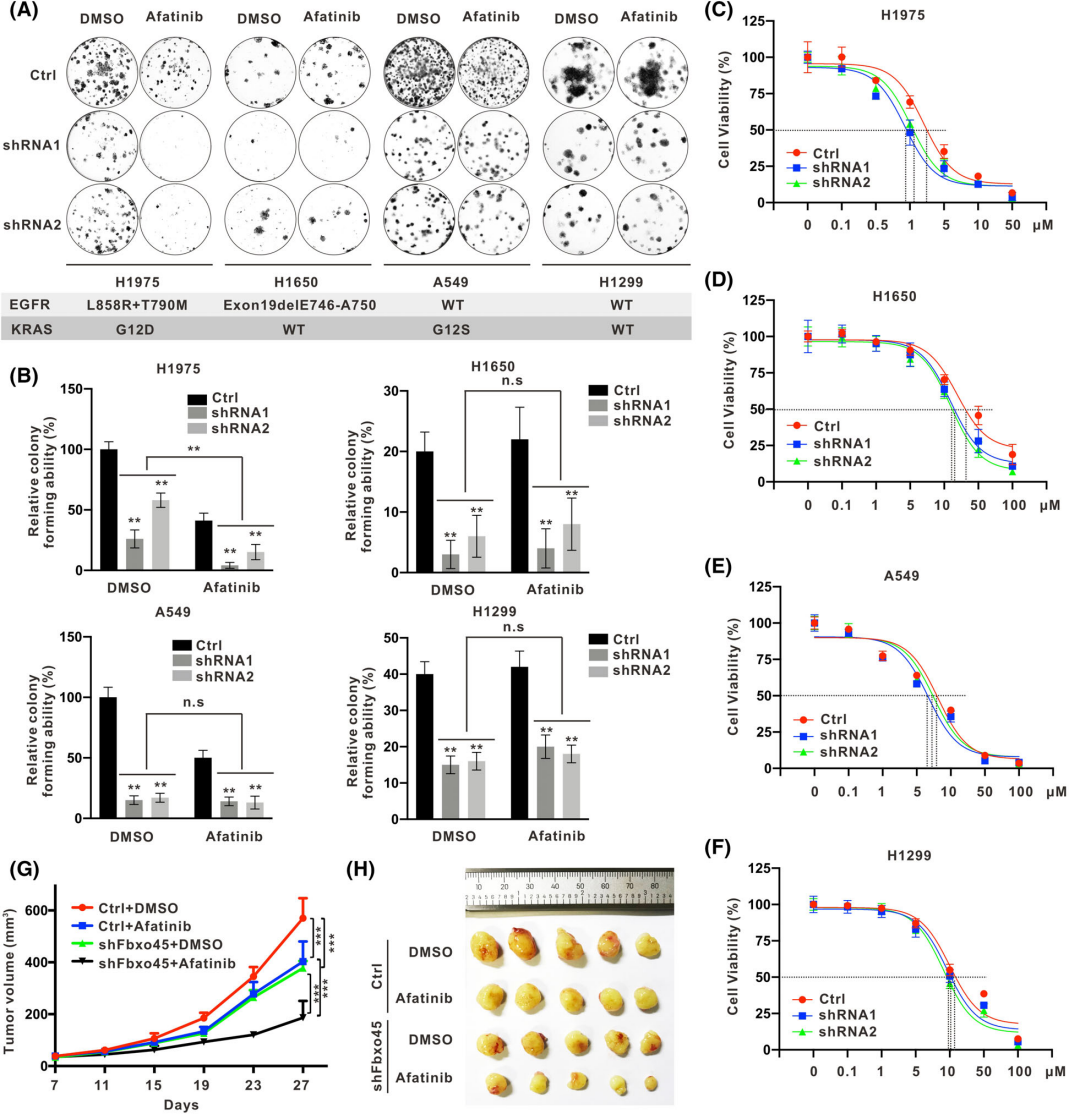
Fbxo45 silencing increases the sensitivity to Afatinib on H1975 *in vitro* and *in vivo*. (A, B) The colony formation of cells harboring control shRNA or Fbxo45 shRNA with triplicates each was treated with or without EGFT‐TKI Afatinib (1 μm) and stained with 0.02% crystal violet in 12‐well plates (A). The status of EGFR and KRAS in each cell line was shown (A). The graphpad prism 8.0 software statistically columned the positive‐stained cell number, and data are presented as mean ± SEM with *t*‐test from three independent experiments. **P < 0.01 (B). (C–F) In four cell lines, cells with or without Fbxo45 shRNA expression were treated with different Afatinib concentrations with triplicates. The cell viability was detected using the CCK8 kit and calculated for IC50 curve making. The data was reported as mean ± SEM. Drug concentrations corresponding to IC50 were indicated with the dash lines. (G, H) H1975 cells harboring Fbxo45 shRNA were harvested individually for subcutaneous xenografted in Balb/c nude mice. After the tumor formation at 10 days, the mice with similar tumor sizes were divided into two groups for intragastric administration with DMSO or Afatinib (10 mg·kg^−1^). The tumor volume was measured every 4 days to generate the growth curve, and data are presented as mean ± SEM with *t*‐test from five tumors of each group. ***P < 0.001 (G). Mice were executed at 27 days post‐injection, and tumors were dissected for photograph (H). [Colour figure can be viewed at wileyonlinelibrary.com]

Afatinib is an irreversible inhibitor of the ErbB family of tyrosine kinases, which is the first‐line treatment for patients with advanced NSCLC as well as patients with brain metastases and activating EGFR mutations [42], including common mutations (Exon19Del/L858R) and uncommon mutations (S768I/G719X/L861Q) [43]. However, resistance to EGF‐TKIs is all along with the clinical challenge [44], such as T790M or/and C797S mutation caused intrinsic resistance, acquired resistance, or adaptive resistance [45, 46]. To assess whether Fbxo45 contributes to Afatinib resistance in NSCLC cells, we exposed four cell lines with abrogating or non‐abrogating Fbxo45 to a low dose of Afatinib (1 μm) for observing the ability of cell colony formation. The results showed that the colony‐forming was significantly reduced in all four cell lines with Fbxo45 silencing no matter with the EGFR or KRAS status and additively reduced in the H1975 cells harboring EGFR‐T790M/L858R and KRAS‐G12D mutations by treating with Afatinib (Fig. 5A,B). According to the drug‐sensitive test, silencing Fbxo45 attenuated the tolerance of H1975 and H1650 cells to Afatinib, with median inhibitory concentration (IC50) change about 2–3 folds, respectively (Fig. 5C,D), but did no or slight effect on that of A549 and H1299 cells (Fig. 5E,F). In addition, Fbxo45 had impact on neither A549 nor H1975 cells regarding the tolerance to the third‐generation EGFR‐TKI Osimertinib (Fig. S1F,G). H1975 cells with abrogating and non‐abrogating Fbxo45 were subsequently transplanted to nude mice. Afatinib treatment elicited a maximum growth inhibition of H1975 cells with Fbxo45 abrogation (Fig. 5G,H and Fig. S1H,I). Together, these indicated that silencing Fbxo45 played additive effects with Afatinib on suppressing the tumor growth of H1975 cells, even though harboring EGFR‐T790M resistance mutation and KRAS mutation, *in vitro* and *in vivo*.

## 4 Discussion

The discovery in 2004 that a subset of NSCLC patients with EGFR‐activated mutations showed a dramatic clinical response to the first‐generation EGFR‐TKI Gefitinib ushered in the era of targeted therapy in NSCLC [47, 48, 49]. Subsequent identification of ALK rearrangements [50] and several cancer‐driver events, including ROS1, NRG1, NTRK1/RET fusion, BRAF (V600E) mutation, or MET amplification/Exon14 skipping, strengthened the concept of oncogene addiction and tumor heterogeneity as a pillar of modern cancer therapeutics [49]. However, with the development and clinical application of targeted therapy, drug resistance of lung cancer patients to EGFR‐TKIs is the most intractable issue in current. The third‐generation EGFR‐TKIs (e.g., Osimertinib and Almonertinib), especially those targeting T790M mutation, have been approved for use in clinical to overcome drug resistance of the first/s EGFR‐TKIs and showed a well therapeutic effect [51, 52, 53]. Nevertheless, it still displays limited clinical efficacy in some NSCLC patients, which results from EGFR‐dependent (e.g., C797S mutation or some rare mutations) or EGFR‐independent (e.g., RAS/BRAF‐V600E mutations, MET/HER2 amplifications, RAS‐MAPK/PI3K‐AKT pathways activation, or oncogenic fusion) mechanism of drug‐resistant [54]. In contrast, Afatinib is active in patients with both common mutations (Del19/L858R) and specific uncommon EGFR mutations (S768I/G719X/L861Q), as well as in patients with nervous metastases, but not against tumors harboring T790M or exon 20 insertions [43, 55]. It is undeniable that Afatinib is yet the first‐line EGFR‐TKI for NSCLC, with an excellent therapeutic effect.

Dysregulation of ERK1/2 signaling is the most frequent alteration in human cancers, including lung cancer [9, 56]. One prominent resistance mechanism to EGFR‐TKIs, KRAS, or MEK inhibitors is the reactivation of ERK1/2 signaling, which can drive cancer cell survival and proliferation [54, 57, 58, 59]. Therefore, blocking the aberrant activation of ERK1/2 seems to be a prospective therapy strategy and a practical approach to combat EGFR‐TKI resistance. The attempts of KRAS and MEK inhibitors are just on such considerations, but the failure of clinical trials has frustrated further research on these treatment strategies [16, 17, 18]. In addition to the kinase cascade, the dephosphorylation of ERK1/2 is also an important pathway to regulate RAS‐MAPK signaling. It contributes to the release of drug resistance in NSCLC [60]. Tohme et al. and Sangodkar et al. have reported that the small molecule activator can reactivate phosphatase 2A (PP2A) to suppress the ERK activity and may be applicable for NSCLC treatment [61, 62]. However, the molecular mechanisms by which some other phosphatase abnormalities lead to aberrant ERK activation in NSCLC remain unclear. We report that Fbxo45 is an ‘intrinsic driver’ of ERK activity maintenance and can cause EGFR‐ or RAS/MAPK‐dependent drug resistance, such as Afatinib (Fig. 5).

Fbxo45 is an estrogen‐induced F‐box protein that inclines to form an atypical ubiquitin E3 ligase complex with MycBP2 (also named PAM) and SKP1 [34, 35, 63]. Increasing evidence has shown that Fbxo45 functions as an oncogene to promote cancer cell survival, tumor growth, and chemotherapy resistance [64, 65, 66]. There is literature proving that Fbxo45 is also associated with the poor prognosis of gastric cancer and degradation of EMT‐related transcriptional factors in breast cancer or prostate cancer [35, 67, 68]. In this work, we reported that Fbxo45 is overexpressed in NSCLC and is closely related to the overall survival of NSCLC patients and the capability to promote tumor cell growth *in vitro* and *in vivo* (Figs 1 and 2). On the mechanism, aberrantly expressed Fbxo45 can sustain the ERK phosphorylation in NSCLC cells by degrading the specific ERK phosphatase STEP according to the analysis in Fbxo45‐silenced or ‐knockout cells (Fig. 3A–E). In addition, we found that nuclear‐localized Fbxo45 can directly interact with STEP_46_ protein, specifically with NP‐STEP_46_, *in vitro* and *in vivo* (Fig. 4C–G). In the ubiquitin‐mediated proteolysis system, the K48‐linkage is a more common poly‐ubiquitin chain in charge of the degradation of substrates, but STEP_46_ was mainly conjugated with K6‐linkage for Fbxo45‐mediated proteasome degradation (Fig. 4H–J). To be mentioned, direct overexpression of Fbxo45 in NSCLC did not further promote ERK phosphorylation levels and relevant biological phenotypes (data not shown). However, restoration of Fbxo45 expression in Fbox45‐silenced or ‐knockout cell lines did (Fig. 3F–H). For this reason, we believe that a small amount of Fbxo45 protein is enough to degrade the activated STEP_46_ completely; therefore, more expressed Fbxo45 will not further affect the ERK activity.

## 5 Conclusions

**Fig. 6:**
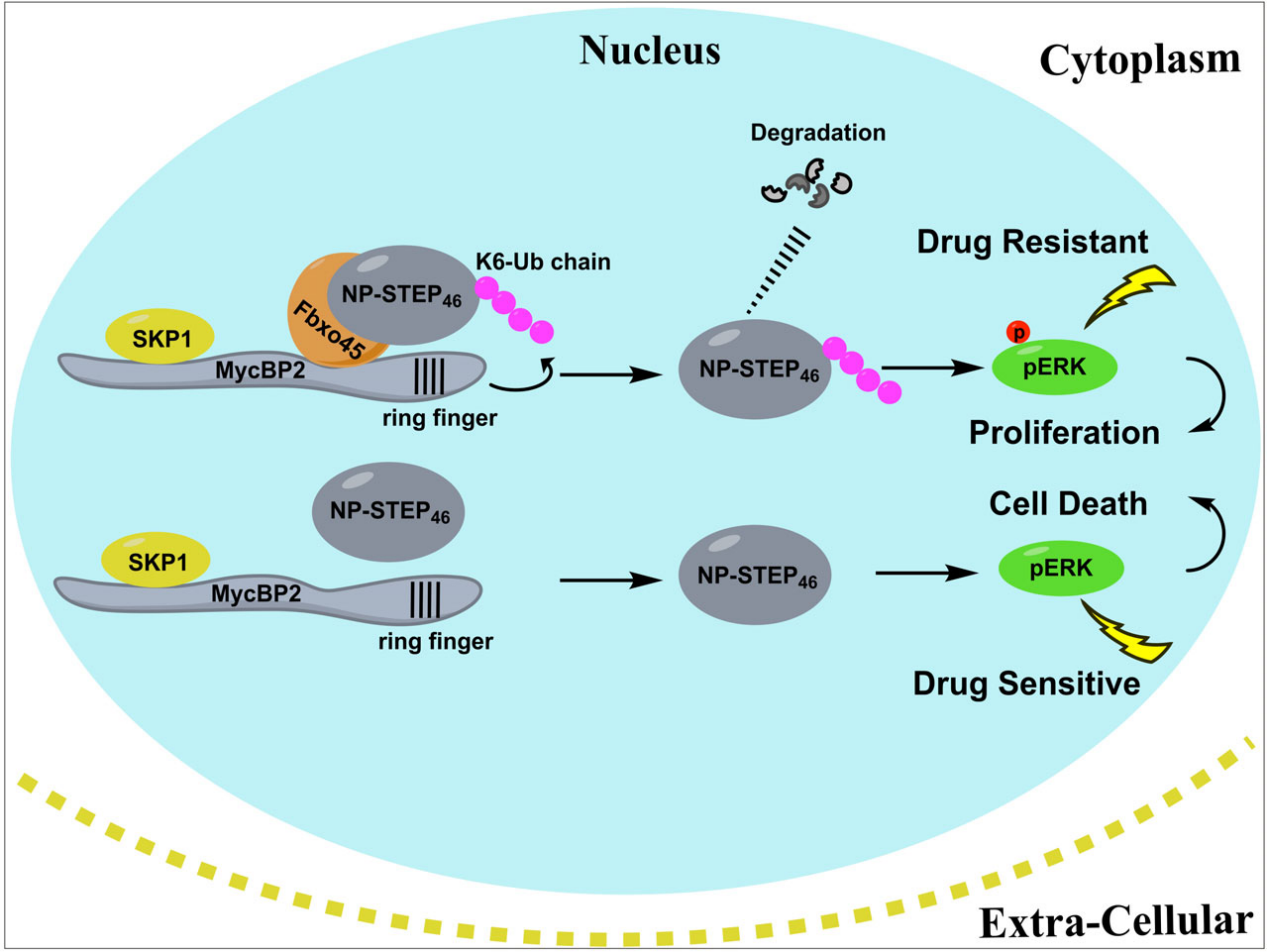
Fbxo45‐mediated NP‐STEP_46_ degradation via K6‐linked ubiquitination sustains ERK activity and drug resistance in lung cancer. The dynamic balance of ERK1/2 activation and inactivation is essential for mammalian cells to maintain normal development, proliferation, differentiation, transformation, and apoptosis. The abnormal activation of ERK1/2 or failure to dephosphorylate pERK1/2 in time is the primary cause of drug resistance in lung cancer cells, a severe EGFR‐targeted therapy issue. Aberrantly expressed Fbxo45 in NSCLC cells can recognize ERK‐specific phosphatase NP‐STEP_46_ (an isoform of activated STEP) and mediate proteasome degradation through conjugating the K6‐ubiquitin linkage on it in the nucleus, resulting in the maintenance of abnormal phosphorylation levels of ERK1/2. On the contrary, inhibiting Fbxo45 prevents NP‐STEP_46_ from degrading efficiently and suppresses the abnormal activation of ERK. Here, we reveal insight into pERK‐maintenance for NSCLC cell survival, proliferation, and drug resistance. Targeting Fbxo45 or combining it with EGFR‐TKIs but not a limit may be an attractive therapeutic strategy to combat the issue of drug resistance in NSCLC patients. [Colour figure can be viewed at wileyonlinelibrary.com]

We depict a novel mechanism that the phosphatase STEP (an active form NP‐STEP_46_) was subjected to proteasome‐mediated degradation in a manner of K6‐linked poly‐ubiquitination caused by the enhanced Fbxo45 expression in NSCLC, which then sustained the ERK1/2 activity for tumor cell survival, tumor growth, and even the drug resistance (Fig. 6). More Fbxo45 functions may exist beyond sustaining ERK1/2, such as cell cycle regulation and inflammatory response, which will be further researched. Finally, our findings provide novel insight into the role of Fbxo45 in NSCLC and may offer an attractive therapeutic target and strategy for this cancer disease. Notably, Fbxo45 is a protein specifically expressed in the nervous system and rarely expressed in other organs and tissues (except tumors), which makes Fbxo45 a promising target for specific drugs to minimize adverse reactions.

## Author contributions

MX designed and guided the whole study, and QW carried out most of the experiments and data collection; QW and CX performed H&E staining and IHC analysis. QW, WA, and RC performed cloning, mutagenesis, and viral infection assays. QW, CX, WA, and RC performed cell phenotype assay and animal experiments. HY collected the clinical data and information. MX and QW wrote and edited the manuscript.

## Conflict of interest

The authors declare no conflict of interest.

### Peer review

The peer review history for this article is available at https://publons.com/publon/10.1002/1878‐0261.13290.

## Supporting information


**Fig. S1.** Fbxo45 is associated with phenotype alteration and drug resistance in NSCLC.

## Data Availability

All the datasets generated and analyzed during this study are available from the corresponding author on reasonable request.
