# Supplementary material for: Fbxo45‐mediated NP‐STEP46 degradation via K6‐linked ubiquitination sustains ERK activity in lung cancer
Source: Mol Oncol. 2022 Aug 5;16(16):3017–33. doi: 10.1002/1878-0261.13290 (PMC9394119; doi:10.1002/1878-0261.13290)
Supplement: Supplementary file 1 — Fig. S1. Fbxo45 is associated with phenotype alteration and drug resistance in NSCLC. [file MOL2-16-3017-s001.zip › MOL2_13290_Supplementary informations.docx]

**Supplementary information**

**Figure Legend**

**Fig.S1 Fbxo45 is associated with phenotype alteration and drug resistance in NSCLC. (a)** The LUAD patients tissue chip, containing 150 samples but only 71 paired due to individual nonadhesive adjacent or tumor tissues on the chip, was detected with Fbxo45 primary antibody (1:50 dilution) and HRP labeled goat anti-rabbit second antibody (1:100). The nucleus was stained with DAPI, and the whole chip was scanned by the Aperio ImageScope system (upper panel). HE staining was shown in the lower panel. **(b)** The subcutaneous xenografted Balb/c nude mice were executed at 27 days post-injection with A549 cell lines with or without Fbxo45 shRNA expression, and the tumor weight was columned for analysis. Data are presented as Mean ± SEM with *t*-test. **p< 0.01. **(c)** The identical tissue chip was detected with pERK primary antibody (1:50 dilution), and HRP labeled goat anti-rabbit second antibody (1:100). The nucleus was stained with DAPI, and the Aperio ImageScope system scanned the whole chip. **(d)** The stable H1299 cell line with overexpressed Flag-STEP_46_ was constructed using the lentiviral system. The cells were harvested after 50 nM EGF stimuli and lysed in RIPA buffer for immunoblotting with indicated antibodies. The experiment was duplicated, and the representative image was reported. **(e)** The proliferation of H1299 cells with or without Flag-STEP_46_ expression was detected using the CCK8 assay kit, and data are presented as Mean ± SEM with *t*-test. ***p< 0.001. **(f-g)** H1975 **(f)** or A549 **(g)** cells with or without Fbxo45 shRNA expression were treated with different concentrations of Osimertinib. The cell viability was detected using the CCK8 kit and calculated for IC_50_ curve making. Drug concentrations corresponding to IC50 were indicated with the dash lines. The data was reported as Mean ± SEM. **(h-i)** H1975 cells harboring Fbxo45 shRNA were harvested individually for subcutaneous xenografted in Balb/c nude mice. After the tumor formation at ten days, the mice with similar tumor sizes in each group were divided into two groups for intragastric administration with DMSO or Afatinib (10mg/kg). The tumor volume **(h)** and weight **(i)** were measured after mice necropsy. Data are presented as Mean ± SEM with *t*-test. *p< 0.05, **p<0.01, ***p<0.001.
