# Supplementary figures and images for: Fbxo45‐mediated NP‐STEP46 degradation via K6‐linked ubiquitination sustains ERK activity in lung cancer
Source: Mol Oncol. 2022 Aug 5;16(16):3017–33. doi: 10.1002/1878-0261.13290 (PMC9394119; doi:10.1002/1878-0261.13290)

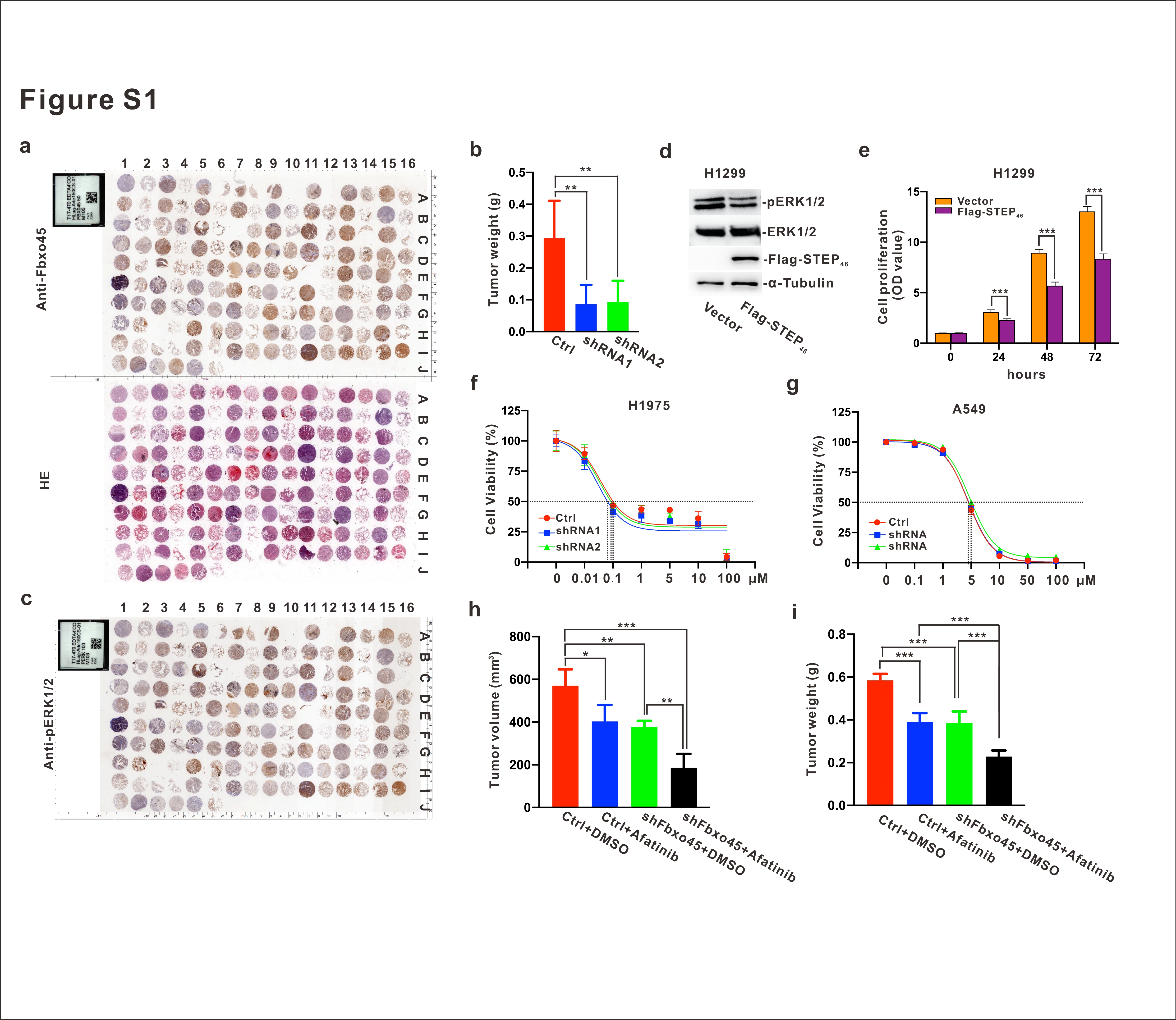

Supplement: Supplementary file 1 — Fig. S1. Fbxo45 is associated with phenotype alteration and drug resistance in NSCLC. [file MOL2-16-3017-s001.zip › MOL2_13290_FigS1.jpg]
